# Comorbidities rather than older age define outcome in adult patients with tumors of the Ewing sarcoma family

**DOI:** 10.1002/cam4.4688

**Published:** 2022-03-16

**Authors:** Jana Käthe Striefler, Maren Schmiester, Franziska Brandes, Anne Dörr, Stefan Pahl, David Kaul, Daniel Rau, Eva‐Maria Dobrindt, Georgios Koulaxouzidis, Lars Bullinger, Sven Märdian, Anne Flörcken

**Affiliations:** ^1^ Charité–Universitätsmedizin Berlin, corporate member of Freie Universität Berlin Humboldt‐Universität zu Berlin Berlin Germany; ^2^ Department of Hematology, Oncology, and Tumor Immunology Berlin Institute of Health Berlin Germany; ^3^ Institute of Pathology, Campus Mitte Berlin Institute of Health Berlin Germany; ^4^ Department of Radiation Oncology Campus Virchow‐Klinikum, Berlin Institute of Health Berlin Germany; ^5^ Centre for Musculoskeletal Surgery Campus Virchow‐Klinikum, Berlin Institute of Health Berlin Germany; ^6^ Department of Surgery Campus Virchow‐Klinikum, Berlin Institute of Health Berlin Germany; ^7^ Department of Plastic, Aesthetic and Reconstructive Surgery, Congregational Hospital Linz Sisters of Mercy Linz Austria; ^8^ German Cancer Consortium (DKTK), partner site Berlin Heidelberg Germany; ^9^ German Cancer Research Center (DKFZ), Heidelberg Germany

**Keywords:** adult patients, Charlson comorbidity index, chemotherapy, Ewing's sarcoma, sarcoma

## Abstract

**Background:**

Ewing family of tumors (EFT) is rarely diagnosed in patients (pts) over the age of 18 years (years), and data on the clinical course and the outcome of adult EFT pts is sparse.

**Methods:**

In this retrospective analysis, we summarize our experience with adult EFT pts. From 2002 to 2020, we identified 71 pts of whom 58 were evaluable for the final analysis.

**Results:**

Median age was 31 years (18–90 years). Pts presented with skeletal (*n* = 26), and extra‐skeletal primary disease (*n* =32). Tumor size was ≥8 cm in 20 pts and 19 pts were metastasized at first diagnosis. Between the age groups (≤25 vs. 26–40 vs. ≥41 years) we observed differences of Charlson comorbidity index (CCI), tumor origin, as well as type and number of therapy cycles. Overall, median overall survival (OS) was 79 months (95% confidence interval, CI; 28.5–131.4 months), and median progression‐free survival (PFS) 34 months (95% CI; 21.4–45.8 months). We observed a poorer outcome (OS, PFS) in older pts. This could be in part due to differences in treatment intensity and the CCI (<3 vs. ≥3; hazard ratio, HR 0.334, 95% CI 0.15–0.72, *p* = 0.006). In addition, tumor stage had a significant impact on PFS (localized vs. metastasized stage: HR 0.403, 95% CI 0.18–0.87, *p* = 0.021).

**Conclusions:**

Our data confirms the feasibility of intensive treatment regimens in adult EFT pts. While in our cohort outcome was influenced by age, due to differences in treatment intensity, CCI, and tumor stage, larger studies are warranted to further explore optimized treatment protocols in adult EFT pts.

## Graphical abstract

Ewing family of tumors (EFT) is rarely diagnosed in patients (pts) over the age of 18 years (yrs). Our data confirms the feasibility of intensive treatment regimens in adult EFT pts. Outcome was influenced by age, due to differences in treatment intensity, CCI, and tumor stage, larger studies are warranted to further explore optimized treatment protocols in adult EFT pts.

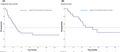

## 1 INTRODUCTION

Although a rare disease, the Ewing family of tumors (EFT) is the second most common primary malignancy originating from bone. In addition to the histologic entities of Ewing sarcoma (ES) and primitive neuroectodermal tumor, EFT also includes extra‐skeletal ES, malignant small round cell tumors of the thoracopulmonary region (Askin tumor), and atypical ES.^1^ Overall, EFT is a rare cancer entity, mainly occurring in younger patients, especially in young adolescents between 10 and 15 years of age. In Germany, the annual incidence is 2.4 per 1 million adolescents and young adults, respectively.^2^ In adults, EFT are even rarer. Published data suggest a rate of only <20% of all ES occurring over the age of 40.^3^ Furthermore, there is evidence that ES in patients ≥41 years tend to be more aggressive as patients may be more likely to show extra‐skeletal and metastatic disease. Additionally, the survival rate in older patients seems to be generally lower.^4^

At the genomic level, EFT is characterized by chromosomal aberrations involving the *Ewing Sarcoma breakpoint region 1* (*EWSR1*) gene on chromosome 22 resulting in hybrid proteins involved in tumorigenesis. Multiple different gene fusions have been reported, which may influence the course of disease and the outcome.^5^, ^6^, ^7^ In accordance, the revised WHO classification of tumors of soft tissue and bone 2020 defines new subgroups of molecular tumors formerly belonging to the EFT group. For instance, CIC‐fused and BCOR‐rearranged sarcomas are now considered as a separate entity (Ewing‐like sarcoma, ELS).^8^, ^9^, ^10^

During the last decades, multimodal therapeutic approaches have improved the survival of young adolescent patients with EFT. The overall 5‐year‐survival rates range from 40 to 60% in patients with localized as well as metastatic disease at first presentation. In spite of no existing international consensus concerning a standardized prognostic score, there are some established risk factors such as metastatic disease, tumor localization, LDH level, age >15 years, tumor size ≥8 cm, and response to neoadjuvant therapy.^11^, ^12^ Furthermore, the body mass index (BMI) and the presence of comorbidities might impact EFT patient outcome.^13^, ^14^

In general, the multimodal therapy approach spans a time of 8–12 months and consists of neoadjuvant chemotherapy, local therapy (surgery and/or radiation), and adjuvant chemotherapy. Treatment standards have only been established for younger patients and rely on results of international trials of which the Ewing 2008 trial has defined the chemotherapeutic standard.^11^ In this trial, in the neoadjuvant setting patients received six cycles of vincristine, ifosfamide, doxorubicin and etoposide (VIDE). After surgery and/or local radiation, patients either received eight cycles of adjuvant chemotherapy with vincristine, actinomycin D, and ifosfamide (VAI) (males) or eight cycles of vincristine, actinomycin D, and cyclophosphamide, (VAC) (females). Recently, the European Ewing 2012 trial (ISRCTN92192408) demonstrated superiority of an induction therapy of vincristine, doxorubicin, and cyclophosphamide alternating with ifosfamide and etoposide (VDC/IE) improving both event free survival and overall survival (OS),^15^ and thus resulting in a refined treatment standard.

Until now, there is only sparse data for the optimal treatment of adults with EFT. The therapeutic approach is most often less intense than respective pediatric therapy protocols. Even with some evidence for the benefit of a very aggressive treatment, practice‐defining studies are still lacking, but there is some data available regarding the feasibility of standard dose intense therapy in the adult patient population.^16^, ^17^, ^18^ Usually, VDC/IE is given to patients with localized disease. In the metastasized situation, vincristine, doxorubicin, actinomycin D, and cyclophosphamide are commonly used.^14^, ^15^, ^16^, ^19^

For relapsed and/or refractory disease, there are two equally effective therapeutic options, topotecan/cyclophosphamide or high‐dose ifosfamide. The combinations of gemcitabine/docetaxel or irinotecan/temozolomide are alternative options, but of inferior efficacy as shown in the rEECur trial.^20^, ^21^ Furthermore, there is emerging data on targeted therapies in EFT,^22^ and relevant effort is put into personalizing therapeutic strategies based on underlying molecular aberrations identified via sequencing individual tumors in scientific programs. One example hereof is the molecularly aided stratification for tumor eradication (MASTER) Program led by the National Center for Tumor Diseases (NCT) in Heidelberg within the German Cancer Consortium (DKTK).^23^ It comprises a central rapid‐turnaround molecular profiling and streamlined data acquisition and analysis of rare cancers, including EFT.

In summary, there is a high unmet medical need to further optimize the treatment of EFT in adult patients, but this can only be done based on an improved understanding of the treatment outcome with current treatment strategies. In accordance, we present here our specialized center experience in adult patients with this rare entity.

## 2 MATERIAL AND METHODS

### 2.1 Patients

In total, 68 EFT and three ELS patients ≥18 years who were newly diagnosed and/or treated at Charité‐Universitätsmedizin Berlin between 2002 and 2020 were identified, of whom 58 were eligible for this analysis. Data were retrospectively extracted from archived patient records.

### 2.2 Treatment

Patients with an adequate performance status (ECOG 0–2) were treated within or on the basis of the Euro EWING 99 and 2008 trials.^11^ Dose adjustments were done on an individual basis, but in‐line with standardized protocols. Patients gave written consent according to institutional and national guidelines. Local treatment consisted of surgery and/or radiotherapy and was individually planned for each patient.

### 2.3 Statistical analysis

OS and progression‐free survival (PFS) were calculated from the date of diagnosis to death or to first event of progression, respectively. For PFS, an event was defined as distant relapse, local relapse, or death, whichever came first. OS and PFS curves were calculated using the Kaplan–Meier method and compared using the log‐rank test and Cox regression with hazard ratios (HR) and 95% confidence intervals (95% CI) as indicated. Chi‐square tests were used to examine associations between categorical variables. A *p* ≤ 0.05 was considered significant. Data analysis was performed using the IBM SPSS Statistics (version 25) software.

## 3 RESULTS

### 3.1 Patient characteristics

Thirty‐three (57%) female and 25 (43%) male patients were included. Median age of the overall cohort at diagnosis was 31 years (range 18–90 years) with 17 (29%) patients being ≥41 years. Median Charlson comorbidity index (CCI)^24^ was 2 (range 2–7), whereas the median BMI was 24.7 (range 16.1–43.4). Median follow‐up was 23 months (range 1–219 months) from diagnosis. Baseline patient characteristics are provided in Table 1.

**TABLE 1:** Baseline patient characteristics

Characteristic	All	≤25 years	26–40 years	≥41 years	*p* value
Age, *n* (%)	58	21 (36)	20 (34)	17 (29)	
Sex, *n* (%)					0.168
Male	25 (43)	8 (38)	12 (60)	5 (29)	
Female	33 (57)	13 (62)	8 (40)	12 (71)	
CCI at diagnosis, *n* (%)					0.003
2	34 (59)	15 (71)	15 (75)	4 (24)	
≥3	24 (41)	6 (29)	5 (25)	13 (76)	
BMI, *n* (%)					0.280
<25	37 (64)	15 (71)	10 (50)	12 (71)	
≥25	21 (36)	6 (39)	10 (50)	5 (29)	

*Note*: Shown are the baseline patient characteristics of all patients as well as of the three different age groups (≤25 years vs. 26–40 years vs. ≥41 years).

Abbreviations: BMI, body mass index; CCI, Charlson comorbidity index.

### 3.2 Tumor localization and size

In our patient cohort, *n* = 26 patients (45%) had a skeletal and *n* = 32 (55%) had an extra‐skeletal primary tumor, respectively (see Table 2). At the time of diagnosis *n* = 19 (33%) had metastatic and *n* = 39 (67%) localized disease. In male patients, appendicular skeleton localization was most common (40%), followed by non‐pelvic (28%), pelvic (24%), and axial (8%) tumor sites. In contrast, non‐pelvic (33%) and axial (30%) localization was most frequent in female patients, followed by appendicular (27%) and pelvic (10%) tumors. An extra‐skeletal primary tumor was less common in male than in female patients (40% vs. 67%), whereas a skeletal origin was more frequent in males than females (60% vs. 33%).

**TABLE 2:** Baseline tumor characteristics

Characteristic	All	≤25 years	26–40 years	≥41 years	*p* value
Primary tumor site, *n* (%)					0.882
Pelvic	9 (15)	5 (24)	3 (15)	1 (6)	
Non‐pelvic	18 (31)	2 (10)	9 (45)	7 (41)	
Axial	12 (21)	4 (19)	3 (15)	5 (29)	
Appendicular	19 (33)	10 (47)	5 (25)	4 (24)	
Tissue origin, *n* (%)					<0.001
Skeletal	26 (45)	15 (71)	10 (55)	2 (12)	
Extra‐skeletal	32 (55)	6 (29)	8 (45)	15 (88)	
Stage, *n* (%)					0.106
Localized	39 (67)	15 (71)	13 (65)	11 (65)	
Distant metastases	19 (33)	6 (29)	5 (35)	6 (35)	
Size, *n* (%)					0.376
<8 cm	15 (43)	7 (54)	4 (29)	4 (50)	
≥8 cm	20 (57)	6 (46)	10 (71)	4 (50)	
Translocation, *n* (%)					0.025
EWSR1 translocation, not further described (EWSR1‐unknown)	21 (55)	11 (86)	4 (27)	6 (60)	
EWSR1‐FLI1	12 (32)	1 (7)	8 (53)	3 (30)	
EWSR1‐ERG	2 (5)	1 (7)	1 (7)	0 (0)	
CIC‐DUX4	3 (8)	0 (0)	2 (13)	1 (10)	

*Note*: Shown are the baseline tumor characteristics of all patients as well as of the three different age groups (≤25 years vs. 26–40 years vs. ≥41 years).

Abbreviations: CIC‐DUX4, Capicua–double homeobox 4; ERG, transcriptional regulator ERG; EWSR1, Ewing sarcoma breakpoint region 1; EWSR1‐FLI1, EWSR1/FLI1 fusion protein type 1.

In patients ≥41 years, a non‐pelvic tumor localization was most commonly seen (41%), followed by an axial (29%), an appendicular (24%), and a pelvic primary (6%). There was a significant association of an extra‐skeletal primary tumor with age ≥41 years (88%; *p* < 0.001). In patients aged 26–40 years, the most common primary tumor site was also non‐pelvic (45%), followed by an appendicular localization (25%). The remaining patients (30%) distributed equally to a pelvic and an axial localization, respectively. Half of the patients (55%) showed a skeletal origin, whereas the other half (45%) had an extra‐skeletal primary. In younger patients (≤25 years), an appendicular localization was most commonly seen (47%), followed by a pelvic (24%), an axial (19%), and a non‐pelvic (10%) primary. A skeletal primary was more common than an extra‐skeletal origin (71% vs. 29%).

Exact tumor size was documented in *n* = 35 cases (60%). Among these, *n* = 20 (57%) were measured ≥8 cm and *n* = 15 (43%) <8 cm. Relating to tumor size, no significant differences between the three age groups were observed. Patients ≥41 years distributed equally to a tumor size ≥8 and <8 cm (50%). For patients in the 26–40 years age group, tumor size was more often ≥8 (71%) than <8 cm (29%). In younger patients (≤25 years), tumor size was more often <8 cm (54%) than ≥8 cm (46%).

### 3.3 Molecular evaluation

Data of molecular diagnostics of the ES gene were available for *n* = 38 patients (66%). Fluorescence in situ hybridization (FISH) was used in 19 patients (51%), and RT‐PCR in *n* = 12 (33%), whereas a combination of both FISH and RT‐PCR was performed in six patients (16%) (for one patient no information concerning the performed analysis was documented). In half of the patients (*n* = 21, 55%), an aberration of EWSR1 with unknown translocation partner (EWSR1‐unknown) was detected. Identification of the translocation partner was possible in the other half (*n* = 17, 45%): EWSR1‐FLI1 (*n* = 12, 32%), CIC‐DUX4 (*n* = 3, 8%), and EWSR1‐ERG (*n* = 2, 5%).

There were no significant differences referring to molecular tumor characteristics between the three age groups. In patients ≥41 years, we predominantly found EWSR1‐unknown translocations (60%). Furthermore, translocations of EWSR1‐FLI1 (30%), as well as CIC‐DUX4 (10%) were detected. Half of the patients in the 26–40 years age group showed EWSR1‐FLI1 translocations (53%), followed by EWSR1‐unknown (27%), CIC‐DUX4 (13%), and EWSR1‐ERG (7%). In patients ≤25 years, there was no CIC‐DUX4 translocation detected. Most common was EWSR1‐unknown (86%), whereas the remaining patients (14%) distributed equally to tumors with translocations of EWSR1‐ERG and EWSR1‐FLI1 (Table 2).

Two patients were also included in the aforementioned MASTER program of the NCT/DKTK for further molecular profiling, and one additional patient was screened for targeted therapy options within the local Charité molecular tumor board program. In one patient we identified an alteration of the CDKN2A gene, as well as mutations in SMARCA2 and ARID1A. In the other two cases BRCAness and amplifications of MYC and CCND1 were found, respectively.

### 3.4 Comorbidities

In our cohort of patients, a significant association between the age at diagnosis and the CCI was observed (*p* = 0.003). In detail, the median CCI was four in the age group ≥41 years, whereas the median CCI was two in patients ≤40 years. However, a higher CCI was not associated with the frequency of chemotherapy dose modifications due to toxicities.

The median BMI of 24.7 observed in the entire cohort had no relevant impact on the outcome. Interestingly, in patients with a BMI <25 a R0 resection was significantly more frequently achieved than in patients with a higher BMI (66% vs. 27%; *p* = 0.031).

### 3.5 First‐line treatment

#### 3.5.1 Neoadjuvant chemotherapy

In general, patients with a tumor size ≥8 cm more frequently received a preoperative chemotherapy than those with smaller lesions (79% vs. 21%, *p* = 0.008). Altogether, in *n* = 31 patients (53%) of the overall cohort a neoadjuvant chemotherapy was performed. In patients ≥41 years of age, preoperative treatment was realized in *n* = 5 cases (29%), whereas *n* = 12 patients (71%) received no neoadjuvant chemotherapy. Half of the patients (*n* = 8, 47%) received a primary resection allowing both histologic diagnosis and therapy, simultaneously. In the other patients, emergency surgery and/or radiation or palliative treatment were primarily performed. In the younger patients of 26–40 years and ≤25 of age, neoadjuvant therapy was also frequently realized (*n* = 10, 53% and *n* = 16, 76%, respectively).

Overall, the most frequently applied regimen in the neoadjuvant setting was VIDE (*n* = 27, 87%), but in patients ≥41 years, the percentage of patients receiving VIDE was somewhat lower (60%) compared to 90% and 94% in the age groups 26–40 and ≤25 years, respectively. Alternatively, few patients received either VDC/IE or doxorubicin/ifosfamide. With a median of six cycles throughout the entire patient population, there was no statistically significant difference in the number of applied cycles between the age groups, but patients ≥41 years received only two to six cycles, whereas in patients ≤40 years, a maximum of nine cycles was given and the majority of patients received six cycles (Table 3).

**TABLE 3:** Baseline therapeutic data

Characteristic	All	≤25 years	26–40 years	≥41 years	*p* value
Radiation, *n* (%)					0.099
Yes	41 (77)	11 (56)	14 (70)	16 (94)	
No	12 (23)	5 (44)	6 (30)	1 (6)	
No of cycles first‐line chemotherapy, *n* (%)					0.013
<6	10 (21)	1 (7)	2 (11)	7 (44)	
≥6	38 (79)	13 (93)	16 (89)	9 (56)	

*Note*: Shown are the baseline therapeutic data of all patients as well as of the three different age groups (≤25 years vs. 26–40 years vs. ≥41 years).

#### 3.5.2 Local treatment strategies

Altogether, *n* = 55 patients (95%) received local therapy of their primary tumor. In *n* = 34 patients (62%) surgery as well as radiation therapy was performed. In *n* = 21 (38%) either surgery (*n* = 17, 31%) or radiation only (*n* = 4, 7%) was implemented into the therapeutic algorithm. There were no significant differences between the three age groups referring to the choice of the local treatment strategy (Table 3).

#### 3.5.3 Adjuvant therapy

Of the overall cohort, *n* = 41 patients (71%) received adjuvant chemotherapy such as VAI (*n* = 14, 34%), followed by VAC (*n* = 9, 22%), VIDE (*n* = 9, 22%), alternating VAC/VAI (*n* = 3, 7%), and VDC/IE (*n* = 3, 7%) or individual concepts such as VCDE, and combination of ifosfamide and doxorubicin or etoposide.

There were significant differences between the age cohorts concerning the choice of the adjuvant regimen (*p* = 0.004). The majority of patients ≥41 years received VIDE (31%), followed by alternating VAC/VAI (23%) and VAI (15%). In the remaining patients in this age cohort (31%) VAC, VCDE, or doxorubicin/ifosfamide was applied. In patients of 26–40 years, VAI was the most common regimen (40%), followed by VIDE (33%), VDC/IE (13%), and ifosfamide/etoposide or VAC (each 7%). In contrast, the younger patients (≤25 years), received either VAC or VAI (each 50%).

The number of adjuvant chemotherapy cycles given was also significantly different relating to the three age groups (*p* = 0.017). The majority of patients ≥41 and 26–40 years received six cycles (≥41 years: median 6, range 2–8 cycles; 26–40 years: median 6, range 1–9 cycles). In contrast, in the age group ≤25 years the majority of patients received eight cycles (≤25 years: median 8, range 1–8).

### 3.6 Consolidating therapy

Fourteen patients (24%) obtained consolidating chemotherapy subsequent to the first‐line treatment. Of those, *n* = 8 (57%) received VAI, *n* = 4 (29%) VAC and *n* = 1 (7%) VIDE and VAC/VAI, respectively. The median number of cycles were five (range 1–8). Moreover, seven patients (12%) received high‐dose chemotherapy with autologous stem cell support as consolidating therapy in a curative intention (≥41 years: *n* = 1; 26–40 years: *n* = 5; ≤25: *n* = 1).

### 3.7 Treatment of recurrent disease

In our cohort, *n* = 26 patients (45%) showed relapse and/or refractory disease.

#### 3.7.1 Systemic therapy

**FIGURE 1:**
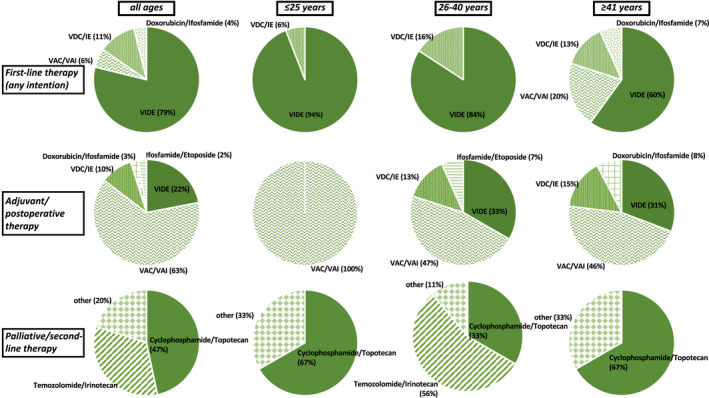
Therapeutic regimen. VAC, vincristine, actinomycin D, and cyclophosphamide; VAI, vincristine, actinomycin D, and Ifosfamide; VDC/IE, vincristine, doxorubicin, cyclophosphamide, and etoposide/ifosfamide; VIDE, vincristine, ifosfamide, dacarbazine, and etoposide. Shown are the applied therapeutic regimen within all patients as well as of the three different age groups (≤25 years vs. 26–40 years vs. ≥41 years) as first‐line, adjuvant/postoperative, and palliative/second‐line therapy, respectively

Data on palliative chemotherapy applied in this situation were available for *n* = 21 patients (81%). The first‐line treatment at relapse most often consisted of temozolomide/irinotecan (*n* = 8, 38%), followed by topotecan/cyclophosphamide (*n* = 7, 33%), and patient‐individual concepts such as topotecan‐ or ifosfamide‐based combinations, as well as DTIC or alternating VAC/VAI. Patients ≥41 years (*n* = 7) received temozolomide/irinotecan (*n* = 4), followed by dacarbazine, cyclophosphamide/topotecan, and vincristine/ifosfamide (*n* = 1 each). Patients aged 26–40 years (*n* = 11) often received cyclophosphamide/topotecan (*n* = 5), temozolomide/irinotecan (*n* = 3), and topotecan‐ or ifosfamide‐based combinations, as well as alternating VAC/VAI (*n* = 1 each). In patients ≤25 years (*n* = 3), temozolomide/irinotecan, ifosfamide, and cyclophosphamide/topotecan was given (*n* = 1 each). The median number of cycles differed among the three age groups (≥41 years: 1, range 0–2; 26–40 years: 2, range 2–10; ≤25 years: 8, range 1–8; see Figure 1 and Table 3).

Second‐line palliative chemotherapy in relapse was applied in *n* = 15 patients (26%) of the overall cohort. Many received cyclophosphamide/topotecan (≥41 years: *n* = 2; 26–40 years: *n* = 3; ≤25 years: *n* = 2), whereas in patients aged 26–40 years temozolomide/irinotecan was also commonly given (*n* = 5; Figure 1).

#### 3.7.2 Local treatment strategies

Altogether, *n* = 13 patients (22%) received radiation therapy in recurrent disease only, whereas in *n* = 4 patients (7%), local irradiation was also performed in the later course of the disease in addition to the prior curatively intended treatment (see Table 3).

### 3.8 Treatment of special cases

In four patients the malignant disease was initially not classified as an EFT, but as a neuroendocrine tumor, a neuroendocrine carcinoma, or an olfactorius neuroblastoma, respectively. In all of those patients, the tumor was primarily resected at an external site. In the majority of cases, the diagnosis was revised in the event of relapse or refractory disease by referral pathology. For instance, systemic therapy consisted of streptozotocin/5‐fluorouracil, as well as folinic acid, 5‐fluorouracil, and oxaliplatin (FOLFOX regimen) combined with radiation therapy.

In addition to the standard molecular diagnostics in EFT, three patients were screened for targeted therapy options within the MASTER program of the NCT/DKTK, and the local Charité molecular tumor board program, respectively. An alteration of the CDKN2A gene was identified, as well as mutations in SMARCA2 and ARID1A. Thus, CDK4/6 inhibition and BET or EZH2 inhibition was recommended. In the other two cases BRCAness and amplifications of MYC and CCND1 were found, respectively. Both patients died due to progressive disease before targeted therapy could be initiated.

### 3.9 Toxicity

Dose modification due to toxicity was performed in *n* = 36 patients (62%) of the overall study population at any time point. The most common reason was the occurrence of both clinical neutropenia and polyneuropathy (*n* = 8, 22%), followed by neutropenia and polyneuropathy as sole symptoms (*n* = 6, 17% each). The combination of both neutropenia and mucositis led to dose reductions in *n* = 4 patients (11%). The remaining dose modifications were due to age >67 years (*n* = 3, 8%), mucositis (*n* = 2, 5%), psychotic symptoms, and/or nephrotoxicity.

During the course of first‐line chemotherapy, a dose modification was observed in 32 patients (55%). Among the three age groups (≥41 vs. 26–40 vs. ≤25 years), no significant difference in the rate of dose modification was found (76% vs. 60% vs. 33%, respectively; *p* = 0.028).

In the palliative therapy setting, dose modifications were documented in *n* = 19 patients (33%). Referring to the frequency of dose reduction, there were no significant differences between the three age groups (≥41: 47% vs. 26–40: 25% vs. ≤25 years: 19).

### 3.10 Outcome

#### 3.10.1 Neoadjuvant therapy

Thirty‐one patients (53%) received neoadjuvant treatment, of whom data on outcome was available for *n* = 28 (90%). In *n* = 18 a CR was detected, whereas *n* = 8 patients achieved PR, and *n* = 2 SD. During neoadjuvant treatment, there was no PD observed. Relating to the different age groups, therapeutic outcome was as follows: ≥41 years: *n* = 3 CR, *n* = 2 PR, *n* = 0 SD; 26–40 years: *n* = 4 CR, *n* = 4 PR, *n* = 2 SD; ≤25 years: *n* = 11 CR, *n* = 2 PR. There were no significant age‐dependent differences.

#### 3.10.2 Postoperative results

Histological evaluation after neoadjuvant therapy and resection was realized in *n* = 21 (36%) of the overall patient population. In the majority of cases (*n* = 15, 71%), a regression grade I according to Salzer–Kuntschik was observed. Two patients had a regression grade II, *n* = 2 grade III, *n* = 1 grade IV, and *n* = 1 grade V after completion of neoadjuvant treatment. Referring to the efficacy of neoadjuvant treatment, there were no significant differences between the three age groups. Data on resection status were available for 42 patients (72%). In *n* = 24 (57%) of these, R0 resection was achieved. R1 resection was documented in *n* = 4 (10%), R2 in *n* = 5 (12%), and Rx in *n* = 9 (21%) cases, respectively. There were no significant differences relating to resection status between the three age groups. Many patients with R0 resection status (*n* = 14, 58%) received local radiation therapy in addition to resection of the primary tumor. There were no significant differences of frequency of adjuvant irradiation referring to resection status. As mentioned above, R0 resection was more frequently observed in patients with a BMI <25 compared to those with a higher BMI (*p* = 0.031).

#### 3.10.3 Completion of first‐line therapy

Altogether, *n* = 41 patients (71%) received adjuvant treatment. Data on outcome after completion of multimodal first‐line therapy were available for 39 patients (67%). In these patients, the clinical staging showed CR in *n* = 29 cases (74%), followed by PD in six patients (15%). In contrast, PR and SD were achieved in *n* = 2 (5%) each. Referring to the three age groups, there were no significant age‐dependent differences observed. The therapeutic outcome was as follows: ≥41 years (*n* = 11): *n* = 7 CR, *n* = 2 PR, *n* = 2 PD; 26–40 years (*n* = 15): *n* = 11 CR, *n* = 2 SD, *n* = 2 PD; ≤25 years (*n* = 13): *n* = 11 CR, *n* = 2 PD. BMI as well as CCI did not have any relevant influence. In patients with a R0 resection as well as with EWSR1‐unknown status CR was more common than in the remaining cases (R0 vs. ≥R0: 62% vs. 38%; *p* = 0.018, and EWSR1‐unknown vs. other translocation: 59% vs. 41%, *p* = 0.005, respectively).

#### 3.10.4 Relapse

Altogether, *n* = 26 patients (45%) showed a relapse of the EFT. Regarding the different age groups, there were no significant age‐dependent differences found. The majority showed further disease progression (*n* = 11, 52%), *n* = 5 (24%) achieved partial remission, *n* = 3 stable disease (14%), and *n* = 2 complete remission (10%), respectively. In detail, the therapeutic outcome was as follows: ≥41 years (*n* = 7): no CR, *n* = 2 PR, *n* = 1 SD, *n* = 4 PD; 26–40 years (*n* = 11): *n* = 2 CR, *n* = 2 PR, *n* = 1 SD, *n* = 6 PD; ≤25 years (*n* = 3): no CR, *n* = 1 PR, *n* = 1 SD, *n* = 1 PD.

Fifteen patients received second‐line palliative treatment (*n* = 1 CR, *n* = 4 SD, and *n* = 10 PD), and *n* = 5 received third‐line palliative treatment (*n* = 2 SD, *n* = 3 PD).

### 3.11 Survival data

**FIGURE 2:**
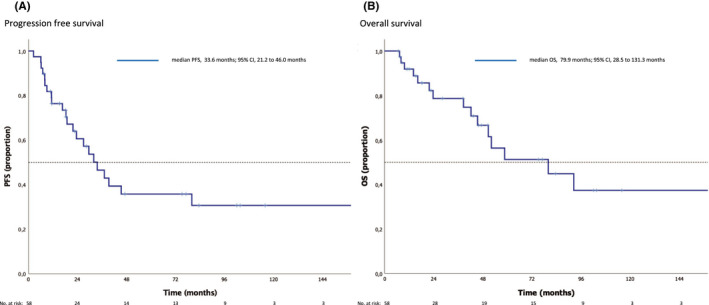
Survival estimates of the overall patient population. Shown are Kaplan–Meier estimates for (A) progression‐free survival, PFS, and (B) overall survival, OS. Median follow‐up of 23 months (range 1–219 months) from diagnosis

Survival parameters (OS, PFS) were evaluable in *n* = 38 (66%) patients. Median OS was 79 months (95% CI 29–131), median progression free survival (PFS) 34 months (95% CI 21–46; see Figure 2).

**FIGURE 3:**
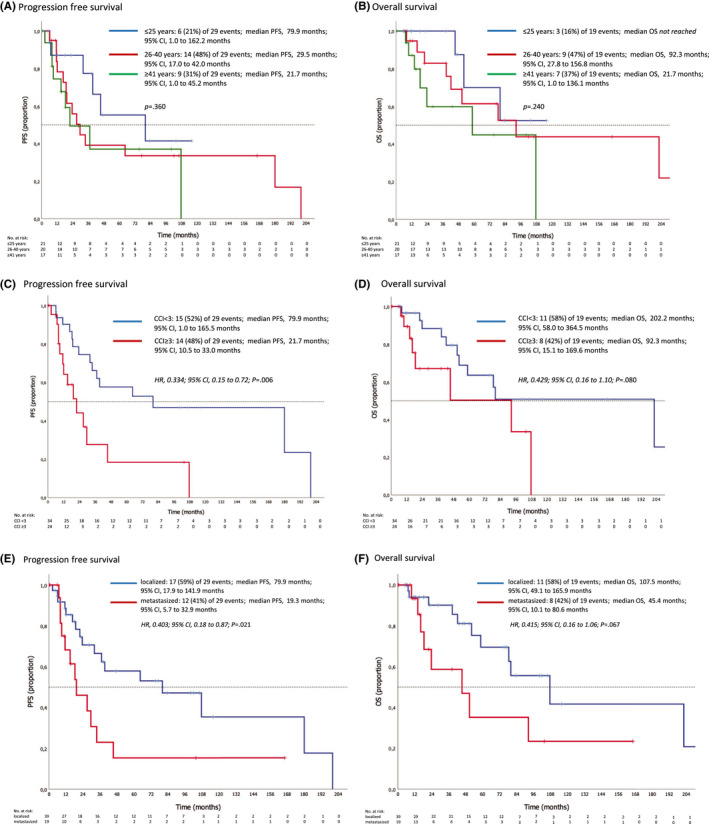
Kaplan‐Meier estimates for progression‐free survival, PFS, and overall survival, OS. (A and B) Age at diagnosis (≤25 years vs. 26–40 years vs. ≥41 years); (C and D) Charlson comorbidity Index, CCI (CCI <3 vs. CCI ≥3); (E and F) Stage at diagnosis (localized vs. metastasized). Shown are Kaplan–Meier estimates for (left) progression‐free survival, PFS, and (right) overall survival, OS. Hazard ratios (HR) derived from univariable Cox regression testing. *p* values derived from log‐rank test. Median follow‐up of 23 months (range 1–219 months) from diagnosis

There was a trend to longer survival in younger patients even if it was not statistically significant neither for PFS nor for OS (median PFS: ≥41 years: 22 months, 26–40 years: 30 months, and ≤25 years: 80 months; median OS: ≥41 years: 59 months, 26–40 years: 92 months, and ≤25 years: not reached), please refer to Figure 3A,B.

Likewise, we observed no significant differences of survival in the curative setting referring to gender and BMI ≥25.

In contrast, univariable analysis showed a relevant influence of CCI on survival. A CCI ≥3 was associated with an impaired OS (HR, 0.429; 95% CI, 0.16–1.10; *p* = 0.080) and PFS (HR, 0.334; 95% CI, 0.15–0.72; *p* = 0.006; see Figure 3C,D).

Tumor stage (localized vs. metastasized) at first diagnosis had a relevant impact on median PFS as well as OS in the curative setting (PFS, HR 0.403, 95% CI 0.18–0.87, *p* = 0.021; OS, HR 0.415, 95% CI 0.16–1.06, *p* = 0.067; see Figure 3E,F). Referring to primary tumor site (pelvic, non‐pelvic, axial, and appendicular) as well as to origin (extra‐skeletal vs. skeletal) there was no relevant difference in survival in the course of first‐line therapy. The diverse genetic aberrations (EWSR1‐unknown, EWSR1‐FLI1, CIC‐DUX, and EWS‐ERG) and tumor size ≥8 cm were not found to impact OS or PFS.

Similarly, for first‐line and/or curative therapy, there were no significant differences in PFS or OS observed relating to the respective regimen, regression grade or resection status as well as to the number of chemotherapy cycles and modalities of local therapy applied.

For second‐line therapy, survival parameters (OS2, PFS2) were evaluable in 20 patients (34%). Median overall survival (OS2) from relapse/progression to death was 17 months (95% CI 7–27), median progression‐free survival (PFS2) calculated from first relapse/progression to second relapse/progression was 8 months (95% CI 3–12). There was no significant difference in PFS2 and OS2, respectively, between the three age groups (≤25 vs. 26–40 vs. ≥41 years). Twenty patients (34%) died due to progression of EFT. There was no therapy‐associated death. A BMI ≥25 had a significant impact on median OS2 (BMI ≥25 vs. BMI <25: 63 vs. 6 months, *p* = 0.013). Other patient characteristics such as age, gender, and comorbidities did not have any significant influence neither on PFS2 nor on OS2. Referring to the tumor characteristics, in particular tumor size, location, as well as tumor stage at first diagnosis, there was no relevant impact on survival in the context of advanced disease and/or palliative therapy observed.

The number of conducted chemotherapy cycles and the regression grade achieved by neoadjuvant treatment significantly influenced PFS2 and/or OS2. In general, median PFS2 was significantly longer in patients receiving more cycles of any first‐line therapy (neoadjuvant, adjuvant, or palliative) than in those who got a smaller number of treatment cycles (*p* = 0.002). In detail, the number of cycles in the neoadjuvant as well as in the adjuvant setting significantly influenced median PFS2: 1 versus 8 months (neoadjuvant therapy: five vs. six cycles; *p* = 0.016), and one versus 10 months (adjuvant therapy: one vs. eight cycles; *p* < 0.001). In addition, the number of adjuvant therapy cycles had a significant impact on median OS2: 5 versus 19 months (one vs. eight cycles; *p* = 0.024). The regression grade according to Salzer–Kuntschik was inversely correlated with the OS2: 5 versus 17 months (regression grade 4 vs. 1; *p* < 0.001). Due to the small sample size, an assessment of the influence of the chemotherapeutic regimen, the resection status as well as of the local therapy on survival was not realizable.

## 4 DISCUSSION

EFT sarcomas are very rare in adult age, therefore most of the literature refers to pediatric patient populations. Thus, our aim was to underline the feasibility of comparatively intense chemotherapeutic regimen in adult patients as well as to examine outcome parameters in our selected single center cohort. In‐line with recently published data, our patient population showed no inferior survival in adult patients in general. However, although not statistically significant, we did observe poorer outcome in patients ≥41 years compared to ≤25 years. In part this can be explained by the comorbidities, which had a relevant influence on outcome. A higher CCI ≥3 was associated with a significant shorter OS and even PFS. This observation is in‐line with previous studies suggesting an adverse effect of comorbidity on survival of sarcoma patients.^25^, ^26^, ^27^ In addition, in accordance with previously published case series, we found a significant higher frequency of extra‐skeletal primary tumors in the older patient which could also explain part of our observation.^3^, ^28^

Furthermore, as expected, patients with metastatic disease had a significantly shorter OS and PFS than those with localized disease. The prominent role of stage in our cohort is consistent with previous analyses in adult patients with EFT and even was described as the sole predictor of survival by Martin II et al.^29^, ^30^, ^31^, ^32^ In addition, some adult patient populations have linked tumor size, gender, and non‐extremity bone location as well as the differing treatment regimen to impact outcome.^29^, ^31^ In contrast, we did not observe respective significant differences related to these specific patient and tumor characteristics. While our study comprised only a small cohort of cases, many of the meta‐analysis or registry data sets might also be biased and this indicates that additional studies need to be performed for patients above the age of 18 years.

For instance, contrary to our expectations a high BMI (≥25) had no impact on any clinical aspect other than survival following relapse and/or progression (OS2). Although Goldstein et al. could show a better survival and an increase of tumor necrosis in their pediatric population with EFT and a normal BMI, we found no significant difference of OS or PFS in our adult cohort depending on body weight.^14^ In general, data on the negative effect of a high BMI on cancer survival is heterogeneous and is not applicable on all cancer types and patient characteristics.^33^ Nevertheless, the comparatively high BMI in our cohort might reflect a selection bias in our patient population or could be indicative that obesity, which has been linked to cancer, might even increase the risk for EFT.

While EFT often present as very aggressive and advanced/metastasized disease, therapeutic concepts are multimodal, combining chemotherapy, surgery, and local radiation ideally. The recent results of the EWING 2012 trial showing a superiority of VDC/IE induction in patients of 5–50 years of age might change the therapeutic approach in EFT.^15^ As previously shown by Lu et al., it is also feasible in the adult EFT patient,^32^ and our data would also support this, as we did not see a general correlation of age with a higher therapy‐ associated toxicity. Of course, based on the long time period of data collection in our study, diagnostic procedures and treatment sequences were heterogeneous, especially in the older patient population, in whom VIDE was less frequently used as primary therapy. This could of course also explain why in general toxicity did not differ among the age groups. Unfortunately, due to the limited quality of the retrospectively acquired data were not able to analyze treatment delays. This important issue might be integrated in subsequent studies.

Interestingly, we also found an association of the number of cycles of any first‐line therapy with the outcome in the relapsed situation (OS2 and PFS2). Patients who received a larger number (≥6) of first‐line treatment cycles showed significantly longer OS2 and PFS2 than those receiving a smaller number (<6). Presumably, the number of cycles of first‐line therapy given might primarily reflect the performance status and/or frailty of the respective patient which both significantly influence the therapeutic possibilities at the time of relapse. On the other hand, this could also be due to a more effective control of minimal residual disease following more treatment cycles. In accordance, there is some evidence for the improvement of survival by use of a high‐dose chemotherapy in selected patients with EFT,^11^, ^34^, ^35^ which could also result in deeper remissions. Patients above the age of 40 might also benefit from this therapeutic approach but are rarely included in the respective trials.

In summary, our study shows the feasibility of an intensive treatment in EFT patients of adult age. In future trials, multimodal treatment approaches will have to be more individually adapted to patient and tumor characteristics. Definition of new molecular subgroups might be the first step on the way to targeted therapeutic strategies. For example, CIC‐fused and BCOR‐rearranged sarcomas are now considered as a separate entity (ELS).^8^, ^9^, ^10^ Whereas, given the absence of a significant impact on survival relating to molecular genetic aberrations as well as the lack of a specific therapeutic strategy for those patients we decided to include them in our analysis.

In the era of increasing availability of molecular genetic diagnostics, a more individualized therapy or even targeting EWSR1‐FLI1 translocations will hopefully be enabled soon.^19^

Additionally, conventional multimodal therapeutic approaches with an optimized therapy intensity and toxicity profile for each patient should also be envisioned. Evidently, there is an urgent medical need to optimize the assessment of elderly patients regarding the increasing gap between chronological and physiological age. Presumably, frailty rather than age might serve as predictor of chemo‐associated toxicity. However, even though a multitude of geriatric assessment tools were developed during the last years, none are routinely integrated into the clinical routine or validated by randomized controlled trials, yet.^36^, ^37^, ^38^

In the absence of a standardized geriatric assessment, decision‐making about the intensity of a multimodal therapy might be based on physiological rather than chronological age taking into account the respective bone marrow reserve, for example. Accordingly, our data suggest that an outcome comparable to younger age groups can also be achieved in older patients.

However, with increasing numbers of long‐term survivors, not only long‐term toxicity, but also the occurrence of secondary neoplasms will have to be taken into account. Thus, as we have already learned from clinical trials in the context of soft tissue sarcoma, we need to move forward from a one‐for‐all strategy toward precision medicine approaches in EFT.

## 5 CONCLUSIONS

With our retrospective analysis, we could confirm a manageable toxicity in adult patients with EFT treated with multimodal therapies. Due to significant differences in the applied first‐line and neo−/adjuvant chemotherapeutic regimen, we could see differences in OS and PFS in the older age group (≥41 years) compared to younger adults (≤25 years). This warrants further exploration, as novel treatment protocols for an optimized multimodal management of patients should be adjusted to the individual age cohorts to ensure the possibility of intensive treatment in all patient groups. This is of great importance, especially as our data demonstrate the influence of comorbidity on outcome, which often might prevent intensive therapy. Furthermore, novel options are needed for advanced tumor stages that have also poor impact on prognosis in EFT. Therefore, therapeutic decisions should not only be based on chronological age, but also on a thorough individual assessment of the patient. Given the rarity of EFT in adults, additional prospective data sets need to be collected in larger cooperative group to allow for further optimization of diagnostic approaches. For example, comprehensive molecular EFT profiling might be a prerequisite for a better understanding of the molecular mechanisms underlying the EFT subgroups with aggressive behavior.

## CONFLICT OF INTEREST

There is no conflict of interest to declare for the author as well as for the coauthors.

## AUTHOR CONTRIBUTIONS

Jana Käthe Striefler had full access to all of the data in the study and took responsibility for the integration of the data and the accuracy of the data analysis. Concept and design: Jana Käthe Striefler and Anne Flörcken. Acquisition, analysis, or interpretation of data: Jana Käthe Striefler, Lars Bullinger, and Anne Flörcken. Drafting of the manuscript: Jana Käthe Striefler, Lars Bullinger, and Anne Flörcken. Critical revision of the manuscript for important intellectual content: Jana Käthe Striefler, Maren Schmiester, Franziska Brandes, Anne Dörr, Stefan Pahl, David Kaul, Daniel Rau, Eva‐Maria Dobrindt, Georgios Koulaxouzidis, Lars Bullinger, Sven Märdian, and Anne Flörcken. Statistical analysis: Jana Käthe Striefler and Anne Flörcken. Administrative, technical, or material support: Jana Käthe Striefler, Lars Bullinger, and Anne Flörcken. All authors have read and approved the manuscript.

## CONSENT FOR PUBLICATION

Not Applicable.

## ETHICS STATEMENT

Our study involving human subjects has been performed with institutional ethical review board approval of Charité's Ethics Committee (Approval Number EA2/240/20) and appropriate participants´ written informed consent in compliance with the Helsinki Declaration.

## Data Availability

All data generated or analyzed during this study are included in this article. Further enquiries can be directed to the corresponding author.

## References

[1] W. C. of T. E. Board . *Soft Tissue and Bone Tumours*. Accessed September 21, 2020. https://publications.iarc.fr/Book‐And‐Report‐Series/Who‐Classification‐Of‐Tumours/Soft‐Tissue‐And‐Bone‐Tumours‐2020

[2] Grünewald TGP , Cidre‐Aranaz F , Surdez D , et al. Ewing sarcoma. Nat Rev Dis Primers. 2018;4(1):5. doi:10.1038/s41572-018-0003-x 29977059

[3] Jahanseir K , Folpe AL , Graham RP , et al. Ewing sarcoma in older adults: a clinicopathologic study of 50 cases occurring in patients aged ≥40 years, with emphasis on histologic mimics. Int J Surg Pathol. 2020;28(4):352‐360. doi:10.1177/1066896919893073 31847636

[4] Karski EE , McIlvaine E , Segal MR , et al. Identification of discrete prognostic groups in Ewing sarcoma. Pediatr Blood Cancer. 2016;63(1):47‐53. doi:10.1002/pbc.25709 26257296 PMC5011751

[5] Zoubek A , Dockhorn‐Dworniczak B , Delattre O , et al. Does expression of different EWS chimeric transcripts define clinically distinct risk groups of Ewing tumor patients? J Clin Oncol. 1996;14(4):1245‐1251. doi:10.1200/JCO.1996.14.4.1245 8648380

[6] Tirode F , Surdez D , Ma X , et al. Genomic landscape of Ewing sarcoma defines an aggressive subtype with co‐association of STAG2 and TP53 mutations. Cancer Discov. 2014;4(11):1342‐1353. doi:10.1158/2159-8290.CD-14-0622 25223734 PMC4264969

[7] Brohl AS , Solomon DA , Chang W , et al. The genomic landscape of the Ewing sarcoma family of tumors reveals recurrent STAG2 mutation. PLoS Genet. 2014;10(7):e1004475. doi:10.1371/journal.pgen.1004475 25010205 PMC4091782

[8] Smith SC , Buehler D , Choi EYK , et al. CIC‐DUX sarcomas demonstrate frequent MYC amplification and ETS‐family transcription factor expression. Mod Pathol. 2015;28(1):57‐68. doi:10.1038/modpathol.2014.83 24947144

[9] Gambarotti M , Benini S , Gamberi G , et al. CIC‐DUX4 fusion‐positive round‐cell sarcomas of soft tissue and bone: a single‐institution morphological and molecular analysis of seven cases. Histopathology. 2016;69(4):624‐634. doi:10.1111/his.12985 27079694

[10] Antonescu CR , Owosho AA , Zhang L , et al. Sarcomas with CIC‐rearrangements are a distinct pathologic entity with aggressive outcome: a clinicopathologic and molecular study of 115 cases. Am J Surg Pathol. 2017;41(7):941‐949. doi:10.1097/PAS.0000000000000846 28346326 PMC5468475

[11] Dirksen U , Brennan B , le Deley MC , et al. High‐dose chemotherapy compared with standard chemotherapy and lung radiation in Ewing sarcoma with pulmonary metastases: results of the European Ewing tumour working initiative of National Groups, 99 trial and EWING 2008. J Clin Oncol. 2019;37(34):3192‐3202. doi:10.1200/JCO.19.00915 31553693 PMC6881099

[12] Hayes FA , Thompson EI , Meyer WH , et al. Therapy for localized Ewing's sarcoma of bone. J Clin Oncol. 1989;7(2):208‐213. doi:10.1200/JCO.1989.7.2.208 2915236

[13] Aggerholm‐Pedersen N , Maretty‐Nielsen K , Keller J , Baerentzen S , Safwat A . Comorbidity in adult bone sarcoma patients: a population‐based cohort study. Sarcoma. 2014;2014:1‐9. doi:10.1155/2014/690316 PMC3958755 24723789

[14] Goldstein G , Shemesh E , Frenkel T , Jacobson JM , Toren A . Abnormal body mass index at diagnosis in patients with Ewing sarcoma is associated with inferior tumor necrosis. Pediatr Blood Cancer. 2015;62(11):1892‐1896. doi:10.1002/pbc.25589 26053354

[15] Bernadette Brennan LK , et al. “Comparison of two chemotherapy regimens in Ewing sarcoma (ES): overall and subgroup results of the Euro Ewing 2012 randomized trial (EE2012).” Accessed July 19, 2020. https://meetinglibrary.asco.org/record/185570/abstract

[16] Cesari M , Righi A , Cevolani L , et al. Ewing sarcoma in patients over 40 years of age: a prospective analysis of 31 patients treated at a single institution. Tumori. 2016;102(5):481‐487. doi:10.5301/tj.5000534 27443894

[17] Fizazi K , Dohollou N , Blay JY , et al. Ewing's family of tumors in adults: multivariate analysis of survival and long‐term results of multimodality therapy in 182 patients. J Clin Oncol. 1998;16(12):3736‐3743. doi:10.1200/JCO.1998.16.12.3736 9850016

[18] Ahmed SK , Robinson SI , Okuno SH , Rose PS , Laack NNI . Adult Ewing sarcoma: survival and local control outcomes in 102 patients with localized disease. Sarcoma. 2013;2013:681425. doi:10.1155/2013/681425 23840168 PMC3693164

[19] Zöllner SK , et al. Ewing sarcoma—diagnosis, treatment, clinical challenges and future perspectives. J Clin Med. 2021;10(8):Art. no. 8. doi:10.3390/jcm10081685 PMC8071040 33919988

[20] McCabe MG , et al. Results of the first interim assessment of rEECur, an international randomized controlled trial of chemotherapy for the treatment of recurrent and primary refractory Ewing sarcoma. J Clin Oncol. 2019;37(15_suppl):11007–11007. doi:10.1200/JCO.2019.37.15_suppl.11007

[21] McCabe MG , et al. Results of the second interim assessment of rEECur, an international randomized controlled trial of chemotherapy for the treatment of recurrent and primary refractory Ewing sarcoma (RR‐ES). J Clin Oncol. 2020;38(15_suppl):11502–11502. doi:10.1200/JCO.2020.38.15_suppl.11502

[22] Italiano A , et al. Cabozantinib in patients with advanced osteosarcomas and Ewing sarcomas: a French Sarcoma Group (FSG)/US National Cancer Institute phase II collaborative study. Ann Oncol. 2018;29:viii753. doi:10.1093/annonc/mdy424.082

[23] Lier A , Penzel R , Heining C , et al. Validating comprehensive next‐generation sequencing results for precision oncology: the NCT/DKTK molecularly aided stratification for tumor eradication research experience. JCO Precis Oncol. 2018;2:1‐13. doi:10.1200/PO.18.00171 35135162

[24] Charlson ME , Pompei P , Ales KL , MacKenzie CR . A new method of classifying prognostic comorbidity in longitudinal studies: development and validation. J Chronic Dis. 1987;40(5):373‐383. doi:10.1016/0021-9681(87)90171-8 3558716

[25] Loong HH , et al. O2‐5‐4 ‐ prevalence and prognostic impact of comorbidities in sarcomas: a population‐based study of 3746 patients in Hong Kong. Ann Oncol. 2019;30:vi88. doi:10.1093/annonc/mdz339.024

[26] Raedkjaer M , Maretty‐Kongstad K , Baad‐Hansen T , et al. The impact of comorbidity on mortality in Danish sarcoma patients from 2000‐2013: a nationwide population‐based multicentre study. PLoS One. 2018;13(6):e0198933. doi:10.1371/journal.pone.0198933 29889880 PMC5995448

[27] Kang S , Kim H‐S , Kim W , Kim JH , Kang SH , Han I . Comorbidity is independently associated with poor outcome in extremity soft tissue sarcoma. Clin Orthop Surg. 2015;7(1):120‐130. doi:10.4055/cios.2015.7.1.120 25729528 PMC4329524

[28] Lynch AD , Gani F , Meyer CF , Morris CD , Ahuja N , Johnston FM . Extraskeletal versus skeletal Ewing sarcoma in the adult population: controversies in care. Surg Oncol. 2018;27(3):373‐379. doi:10.1016/j.suronc.2018.05.016 30217290

[29] Verma V , Denniston KA , Lin CJ , Lin C . A comparison of pediatric vs. adult patients with the ewing sarcoma family of tumors. Front Oncol. 2017;7. doi:10.3389/fonc.2017.00082 PMC5421143 28534008

[30] Martin RCG II . Adult soft tissue Ewing sarcoma or primitive neuroectodermal tumors: predictors of survival? Arch Surg. 2003;138(3):281. doi:10.1001/archsurg.138.3.281 12611575

[31] Baldini EH , Demetri GD , Fletcher CDM , Foran J , Marcus KC , Singer S . Adults with Ewing's sarcoma/primitive neuroectodermal tumor. Ann Surg. 1999;230(1):79‐86. 10400040 10.1097/00000658-199907000-00012 PMC1420848

[32] Lu E , Ryan CW , Bassale S , Lim JY , Davis LE . Feasibility of treating adults with Ewing or Ewing‐like sarcoma with interval‐compressed vincristine, doxorubicin, and cyclophosphamide alternating with ifosfamide and etoposide. Oncologist. 2020;25(2):150‐155. doi:10.1634/theoncologist.2019-0532 32043790 PMC7011630

[33] Greenlee H , Unger JM , LeBlanc M , Ramsey S , Hershman DL . Association between body mass index (BMI) and cancer survival in a pooled analysis of 22 clinical trials. Cancer Epidemiol Biomarkers Prev. 2017;26(1):21‐29. doi:10.1158/1055-9965.EPI-15-1336 27986655 PMC5370550

[34] Ferrari S , Sundby Hall K , Luksch R , et al. Nonmetastatic Ewing family tumors: high‐dose chemotherapy with stem cell rescue in poor responder patients. Results of the Italian Sarcoma Group/Scandinavian Sarcoma Group III protocol. Ann Oncol. 2011;22(5):1221‐1227. doi:10.1093/annonc/mdq573 21059639

[35] Tenneti P , Zahid U , Iftikhar A , et al. Role of high‐dose chemotherapy and autologous hematopoietic cell transplantation for children and young adults with relapsed Ewing's sarcoma: a systematic review. Sarcoma. 2018;2018:1‐12. doi:10.1155/2018/2640674 PMC6008812 29973774

[36] Dale W , Williams GR , MacKenzie AR , et al. How is geriatric assessment used in clinical practice for older adults with cancer? A survey of cancer providers by the American Society of Clinical Oncology. JCO Oncol Pract. 2021;17(6):336‐344. doi:10.1200/OP.20.00442 33064058 PMC8462667

[37] Korc‐Grodzicki B , Holmes HM , Shahrokni A . Geriatric assessment for oncologists. Cancer Biol Med. 2015;12(4):261‐274. doi:10.7497/j.issn.2095-3941.2015.0082 26779363 PMC4706523

[38] Versteeg KS , Konings IR , Lagaay AM , van de Loosdrecht AA , Verheul HMW . Prediction of treatment‐related toxicity and outcome with geriatric assessment in elderly patients with solid malignancies treated with chemotherapy: a systematic review. Ann Oncol. 2014;25(10):1914‐1918. doi:10.1093/annonc/mdu052 24569912

